# Re: Metastatic deaths in retinoblastoma patients treated with intraarterial chemotherapy (ophthalmic artery chemosurgery) worldwide

**DOI:** 10.1186/s40942-018-0124-0

**Published:** 2018-05-29

**Authors:** Sameh E. Soliman, Helen Dimaras, Brenda Gallie, Furqan Shaikh

**Affiliations:** 1 The Department of Ophthalmology and Vision Sciences, The Hospital for Sick Children, 555 University Avenue, Room 7265, Toronto, ON M5G 1X8 Canada; 2 Ophthalmology Department, Faculty of Medicine, University of Alexandria, Alexandria, Egypt; 3 The Division of Hematology/Oncology, Department of Pediatrics, The Hospital for Sick Children, Toronto, ON Canada

Dear Editor,

We read with interest the recent Commentary by Abramson and colleagues, entitled “Metastatic deaths in retinoblastoma patients treated with intraarterial chemotherapy (ophthalmic artery chemosurgery) worldwide” [1]. The authors retrospectively collected data on all patients treated with intra-arterial chemotherapy (IAC) between May 2006 and November 2016 from six retinoblastoma centers, in order to determine the rate of metastatic death in patients who received IAC. The six centers collectively treated 1177 eyes of 1139 patients. Three patients died from metastatic retinoblastoma, all from a single center in Argentina, and all associated with refusal of enucleation or poor follow-up. The authors concluded that the rate of metastatic death after IAC conducted at centers with expertise is low, at < 1%. The authors are to be congratulated for their efforts in assembling such a large pooled database of patients with a rare disease, and for determining this reassuring result. However, we would like to point out some shortcomings of their publication.

An important goal of treating retinoblastoma is to prevent tumor spread or metastasis which is a concern with IAC as a treatment for retinoblastoma as it provide good local control without systemic control [2]. Treatment of metastases is an immense burden with the morbidity and lifelong consequences of intensive chemotherapy, autologous stem cell transplantation, and possibly external beam radiation therapy. In developing countries where stem cell transplant is not available, metastatic retinoblastoma is largely incurable [3]. The Commentary does not mention the rate of metastases in 1139 patients and focuses on the single outcome of metastatic *deaths*. Death is, of course, the most important outcome in any trial, but is not the only one of importance.

Both metastases and deaths occur after some time from therapy, and under-detection of delayed events affects the validity of cancer studies. This is especially true for retrospective studies from tertiary or elite referral centers, which often receive international or distant patients for treatment, who then return home and are not longitudinally followed and might have died unreported [4]. Statistical methods, such as Kaplan–Meier analysis where patients are censored at date last seen can adjust for this differential follow-up effect. Was the follow-up duration and quality sufficient to detect most events? How do the centers retrieve data from lost follow-up patients? The Commentary does not provide any time-to-event analysis, nor even a simple median follow-up time.

The duration of follow-up is crucial and longer follow-up often reveals additional events. The Commentary itself provides a noteworthy example of this very point. It states that “a year ago our centers in New York, Philadelphia, Argentina and Switzerland reported on 634 cases with only one metastatic death.” [5] In the Commentary, they report three metastatic deaths from the center in Argentina, suggesting that two additional deaths occurred in 18 months.

The IAC literature has been reported across several scores of publications, each with a different subset of patients, different measurements, and different outcomes [5–17]. This multiplicative piecemeal reporting creates shortcomings in the scientific evidence and inconsistency of numbers presented. A representative example is in the Commentary. The authors state that their six centers treated 1177 eyes of 1139 patients, representing all patients treated with IAC in the included time period. By mathematical necessity, this would mean that no more 38 patients were treated bilaterally. However, the authors from one center have separately published [16] the results of 60 patients treated with IAC bilaterally in 120 eyes from within the same period.

We close by stating that these points should not be seen as controversial, provocative, or adversarial. There is really no controversy in stating that studies should have important outcomes, adequate follow-up, accurate detection, and consistent information. These are bedrock principles and required standards of all clinical studies.
